# Mapping of oxidative stress responses of human tumor cells following photodynamic therapy using hexaminolevulinate

**DOI:** 10.1186/1471-2164-8-273

**Published:** 2007-08-13

**Authors:** Lina Cekaite, Qian Peng, Andrew Reiner, Susan Shahzidi, Siri Tveito, Ingegerd E Furre, Eivind Hovig

**Affiliations:** 1 Department of Tumor Biology, Rikshopitalet – Radiumhospitalet Medical Center, 0310 Oslo, Norway; 2 Department of Pathology, Rikshopitalet – Radiumhospitalet Medical Center, 0310 Oslo, Norway; 3 State Key Lab for Advanced Photonic Materials and Devices, Fudan University, Shanghai, P.R. China

## Abstract

**Background:**

Photodynamic therapy (PDT) involves systemic or topical administration of a lesion-localizing photosensitizer or its precursor, followed by irradiation of visible light to cause singlet oxygen-induced damage to the affected tissue. A number of mechanisms seem to be involved in the protective responses to PDT, including activation of transcription factors, heat shock proteins, antioxidant enzymes and apoptotic pathways.

**Results:**

In this study, we address the effects of a destructive/lethal hexaminolevulinate (HAL) mediated PDT dose on the transcriptome by using transcriptional exon evidence oligo microarrays. Here, we confirm deviations in the steady state expression levels of previously identified early defence response genes and extend this to include unreported PDT inducible gene groups, most notably the metallothioneins and histones. HAL-PDT mediated stress also altered expression of genes encoded by mitochondrial DNA (mtDNA). Further, we report PDT stress induced alternative splicing. Specifically, the ATF3 alternative isoform (deltaZip2) was up-regulated, while the full-length variant was not changed by the treatment. Results were independently verified by two different technological microarray platforms. Good microarray, RT-PCR and Western immunoblotting correlation for selected genes support these findings.

**Conclusion:**

Here, we report new insights into how destructive/lethal PDT alters the transcriptome not only at the transcriptional level but also at post-transcriptional level via alternative splicing.

## Background

Photodynamic therapy (PDT) combines a light-activated drug with non-thermal light to cause selective damage to the target tissue [1]. The major mechanism of action of PDT has been shown to be induction of oxidative stress [2,3]. It has also been shown that PDT-mediated oxidative stress induces a transient increase in the early response genes FOS, JUN, MYC, and EGR1 [4,5], heat shock proteins (HSPs) [6-9], as well as SOD2, LUC7A, CASP8, and DUSP1 [10]. Furthermore, relevant information exists regarding specific gene expression patterns regulated by oxidative stress [5,11-18]. Signaling pathways influenced by PDT have not been fully elucidated, although a number of studies have addressed this issue [5,10,19]. Moreover, relatively little is known regarding global gene activity, particularly when oxidative stress becomes excessive, as is the case for PDT. Both clonogenic survival of cells from tumors after *in vivo *PDT treatment [20] and resistance to aminolevulinic acid (ALA)-mediated PDT [21] have been reported previously. Intrinsic cell sensitivity to PDT has been proposed [20] to be an important component in the mechanism that leads to tumor response following PDT treatment *in vivo*.

A better understanding of the mechanics of the destructive PDT could facilitate further the development of this therapy. Oxidative stress evokes many intracellular events including apoptosis [22]. Modulating the anti-apoptosis factors that are activated by survival signaling may improve efficacy of the therapy. Under conditions where oxidative stress is the initiating stimulus for apoptosis, it is assumed to simply trigger cell death as a result of cumulative oxidative damage. However, accumulating evidence now suggests that reactive oxygen species (ROS) may act as signaling molecules for the initiation and execution of the apoptotic death program in many, if not all, current models of apoptotic cell death [23,24]. Signaling by ROS would not appear to be random, as previously assumed, but targeted at specific metabolic and signal transduction cellular components [25].

Here, we address the effects of a destructive/lethal PDT dose on the transcriptome by using transcriptional exon evidence oligo microarrays. This dose induces high levels of cytotoxicity and is expected to have significant impact on gene expression patterns. The expression alterations were observed by investigating both early responses, and responses post mobilization of major response pathways. We show that high levels of cellular cytotoxicity have a direct effect on cellular transcription levels and impair metabolic processes. Alternative splicing represents a key event in the control of gene expression [26-30]. Here, we tested to what extent mitochondrial damage caused by HAL-PDT modulates alternative splicing in a global manner.

## Results and discussion

### Rationale for selection of experimental parameters

#### Sensitizer

5-Aminolevulinic acid (ALA), a precursor to porphyrins, is effective and widely used for PDT of a number of diseases [31-34]. However, a significant shortcoming of ALA is its limited ability to cross certain biological barriers (e.g. cellular membranes), probably due to its low lipid solubility. In contrast, ALA esters are more lipophilic and pass more easily through biological membranes than ALA itself. Hexaminolevulinate (HAL) is a hexyl ester of ALA with a higher lipophilicity. HAL has been shown to be 50–100 times more efficient than ALA at inducing cellular porphyrin formation with a high selectivity [35], and has been approved by the European Union for photodetection of bladder cancer. Thus, HAL is a promising compound for PDT therapy. On this basis, it is reasonable to assume that the mechanisms of action of ALA and HAL are comparable, and that gene expression profiles should be very similar. Therefore, only HAL was chosen for this study.

#### Cell line

Studies are now in progress to test the use of PDT for several types of pre-cancerous conditions and cancer, including cancers of the skin, cervix, bladder, prostate, bile duct, pancreas, stomach, brain, head and neck, as well as lymphoma. PDT using topically applied ALA was first reported for the treatment of cutaneous T-cell lymphoma (CTCL) in 1994. Since then, there have been several reports of its usefulness in treating this disease [33,36-39]. The role of PDT in CTCL to date has been in the treatment of individual patches, plaques, and tumors that have not responded to the other forms of skin directed therapy. While several PDT protocols have been evaluated, further studies remain in order to define the optimal use of PDT in treating patients with CTCL. Moreover, the use of PDT for *ex vivo *purging of autologous bone marrow graft has been proposed [40]. High-dose chemotherapy supported by hematopoietic stem cells transplantation (HSCT) presents an effective way to cure lymphoma/leukemia. Although autologous HSCT has several advantages over allogenous HSCT, autografts may harbor residual occult malignant cells that can cause a tumor relapse after being reinfused to the patient. It is therefore desirable to remove the residual neoplastic cells from the autograft before being applied. The ability of malignant cells to selectively accumulate photosensitizers offers the possibility of using PDT in bone marrow purging. Recently, a study of the purging effects of PDT in leukemic cells mixed with normal bone marrow MNCs was published [41].

Here, we aimed to map general responses on gene expression after PDT therapy. Therefore we employed the Jurkat human T-cell leukemia cell line that previously has been used in multiple PDT-studies. The induction of apoptosis through translocation of apoptosis-inducing factor [42,43] and caspase-3-like activation has been demonstrated for this cell line. Also, other sensitizers in PDT therapy have been shown to induce apoptosis in Jurkat cells [32,44-46].

#### Dose and time course

The aim of this study was to determine the gene expression state of the cell in response to PDT immediately prior to and post switching to the programmed cell death. Here, we measured gene signatures that were likely to represent a combination of both protective and pro-apoptotic signals. The characterization of the transcriptome at early stages (1 h and 2 h) was chosen to reveal the state of the cell prerequisites to the execution of the cell death program.

It has been previously demonstrated that the appearance of cytoplasmic mRNA degradation products occur 4–8 h after induction of apoptosis, independent of the apoptotic signal and the cell line used [47]. The purity and integrity of the RNA are critical for the overall success of RNA-based analysis, including gene expression profiling. For this reasons, the late (4 h) time point was selected to provide an insight into the later stages of this process, but still retain a sufficient numbers of viable cells and RNA integrity necessary for the technical execution of the analysis. In this study, the integrity of total RNA was measured by microcapillary electrophoresis and the degree of degradation was determined (data not shown). With the starting amount of cells kept equal across experiments, a time dependent decrease of the total amount of isolated total RNA was obtained. No compromise in the integrity of the RNA was observed. This indicates the effective removal of the dead cell fraction by the wash/centrifugation step, following degradation of leaked RNA.

**Figure 1:**
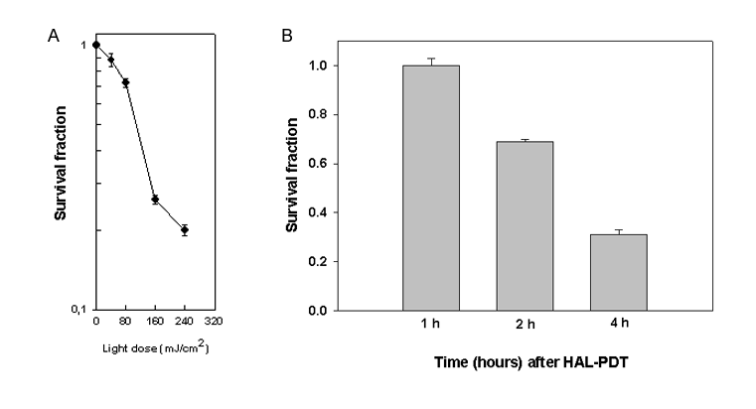
Survival fraction of cells was measured at 4 h after PDT when irradiation was performed with different light doses (A). Cell survival was measured at 1, 2 and 4 h after PDT with the light dose of 160 mJ/cm^2 ^(B). Bars are SE based on three independent experiments with 3 parallels in each.

PDT is a cytotoxic therapy approach, where the dose in therapeutic settings is selected to kill 100% of the target cells. Therefore, a lethal dose (LD) relevant to therapeutic settings was chosen for the study. Lethal and sub lethal doses have been employed in a number of reports that investigated the apoptotic pathways induced by PDT [42,43,48]. The cell survival after HAL-PDT was determined to select the killing dose specific for the Jurkat cell line. Fig. 1A shows the decreased cell survival with increased light doses. Approximately 90% of the cells were killed at 4 h post PDT with the light dose 240 mJ/cm^2^, while 96% of the cells treated with light alone and 98% of the cells treated with HAL alone were still viable (data not shown). The light dose of 160 mJ/cm^2 ^that corresponds to LD 75% at 4 h post HAL-PDT was selected for microarray experiments. The viability of cells treated with LD 75% dose was measured at 1, 2 and 4 h after HAL-PDT (Fig. 1B). There was no change in cell viability observed at 1 h, while the viable cell fraction was reduced to 70% and 30% at 2 h and 4 h post treatment.

### Overview of transcriptome changes

**Figure 2:**
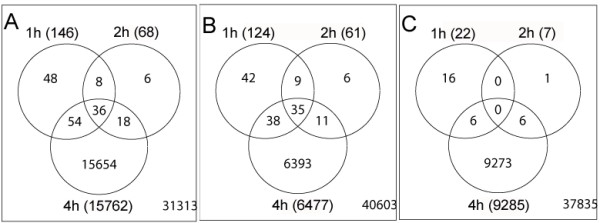
Venn diagrams of differentially altered genes/probes in response to HAL-PDT. A, includes up-regulated and down-regulated; B, only up-regulated; C, only down-regulated genes/probes at 1, 2 and 4 h time course. Each circle represents one time point as indicated. A total number of differentially expressed genes in the group are given in the parentheses. The intersections indicate numbers of genes/probe that are shared between the different time points, i.e. the genes that were steady induced. The number in the right bottom of each square indicates a total post processed and analyzed number of genes/probes minus the number of differentially expressed genes presented in circles.

For gene expression analysis, Jurkat cells were treated with HAL-PDT, and total RNA was harvested from triplicate samples of non-treated and treated cells at 1, 2 and 4 h after HAL-PDT. Gene expression levels in HAL-PDT treated cells relative to non-treated cells were determined by oligo microarrays that contained a total of 44,308 oligos targeting human genes. The Venn diagram shows that 146 probes (103 unique Entrez gene IDs) exhibited significant changes (p < 0.05) 1 h after HAL-PDT treatment (Fig. 2A). The number of significantly altered probes/genes was reduced to 68 probes (53 unique Entrez gene IDs) at 2 h post treatment, with a subsequent large increase at 4 h, with 15,762 probes (10,373 unique Entrez gene IDs). The large number of genes with altered expression at the 4 h time point might reflect a high degree of disturbance in transcriptome within the pro-apoptotic cell. Therefore these genes will be discussed separately as late response genes.

### Early responses

A total of 170 probes (124 unique Entrez gene IDs) were altered at early (1 and 2 h) response (Fig. 2A). Of these 82.9% were up-regulated, *i.e*. 124 probes at 1 h and 61 probes at 2 h (Fig. 2B), while a total of 29 probes were identified as down-regulated at early response (Fig. 2C). Previously, we have reported on the stimulatory effects on the transcriptome using a related treatment modality, photochemical inhibition treatment (PCI) [49]. PCI is based on photosensitizers with photochemical properties similar to those utilized in PDT. However, a weaker dose (LD 50%) is generally used, as the aim is not primarily to cause general cell toxicity. Interestingly, when the lethal PDT dose applied in the present study was compared to those induced by PCI, several of the early altered genes were identified as up-regulated with both modalities, indicating that both moderate (PCI) and high level (PDT) of oxidative stress are likely to invoke similar modulatory pathways, albeit with very different effects at later time points.

**Figure 3:**
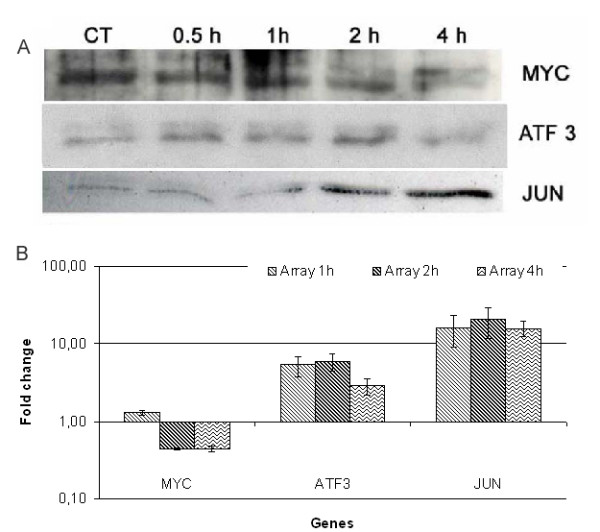
Verification of MYC, ATF3 and JUN expression after HAL-PDT by Western blotting. Cell extracts were prepared at at 1 h, 2 h and 4 h after HAL-PDT (A). Since PDT affected the expression of well-known house keeping proteins such actin (induced) and tubulin (repressed) the total protein content was measured and the gels were stained after electrophoresis, thus normalize against total protein content. The fold-changes obtained by the microarray analysis for the selected genes are presented for comparison. Fold-change of three repeated experiments presented as the mean ± St. Dev (B).

Of the total of 170 probes detected as altered at the two early time points, 44 probes (32 unique Entrez gene IDs) were identified as shared for both the 1 h and 2 h time points in response to HAL-PDT. Among the early response genes, mostly genes encoding proteins involved in the regulation of transcription processes were induced, such as v-jun sarcoma virus 17 oncogene homolog (avian) (JUN), jun B proto-oncogene (JUNB), v-maf musculoaponeurotic fibrosarcoma oncogene homolog B (avian) (MAFB), RNA-binding region (RNP1, RRM) containing 2 (RNPC2), SRY (sex determining region Y)-box 4 (SOX4), tribbles homolog 3 (Drosophila) (TRIB3), Kruppel-like factor 6 (KLF6), zinc finger protein 184 (Kruppel-like) (ZNF184), activating transcription factor 3 (ATF3), BTG family, member 2 (BTG2). While inhibitor of DNA binding 3, dominant negative helix-loop-helix protein (ID3) and v-myc myelocytomatosis viral oncogene homolog (avian) (MYC) were down-regulated. Consistent with gene expression data, the protein products of MYC were found to be reduced, while JUN had increased levels at 2 h and 4 h after HAL-PDT (Fig. 3). Over-expression of ATF3 was seen until 2 h following PDT, but the protein level appears to be decreased at 4 h, probably due to a short half-life (about 30–60 min) of ATF proteins [50]. Several genes belonging to gene ontology group of negative regulation of cell cycle were up-regulated, such as cyclin-dependent kinase inhibitor 1B (p27, Kip1) (CDKN1B), protein phosphatase 1, regulatory (inhibitor) subunit 15A (PPP1R15A) and sestrin 2 (SESN2). Genes coding for proteins of apoptosis regulation were also found to be up-regulated, such as heat shock 70 kDa protein 1B (HSPA1B), tumor necrosis factor (TNF superfamily, member 2) (TNF), tribbles homolog 3 (Drosophila) (TRIB3), protein phosphatase 1, regulatory (inhibitor) subunit 15A (PPP1R15A), nuclear factor of kappa light polypeptide gene enhancer in B-cells inhibitor, alpha (NFKBIA).

#### Histone related genes

**Figure 4:**
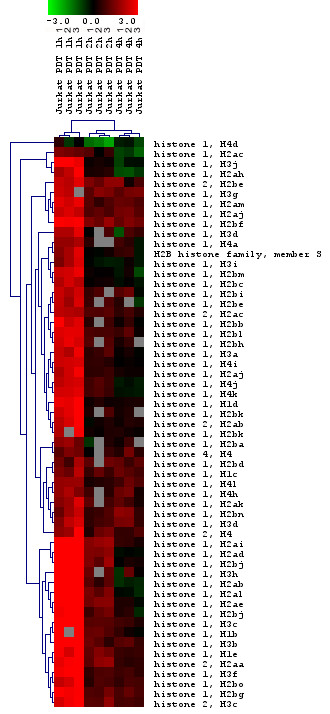
Cluster of histone genes that were significantly altered in response to HAL-PDT. Horizontal stripes represent genes and columns show treatment protocols. The log2-fold changes of gene ratios are color coded as shown in the bar. The three different columns represent repeated microarray experiments.

**Figure 5:**
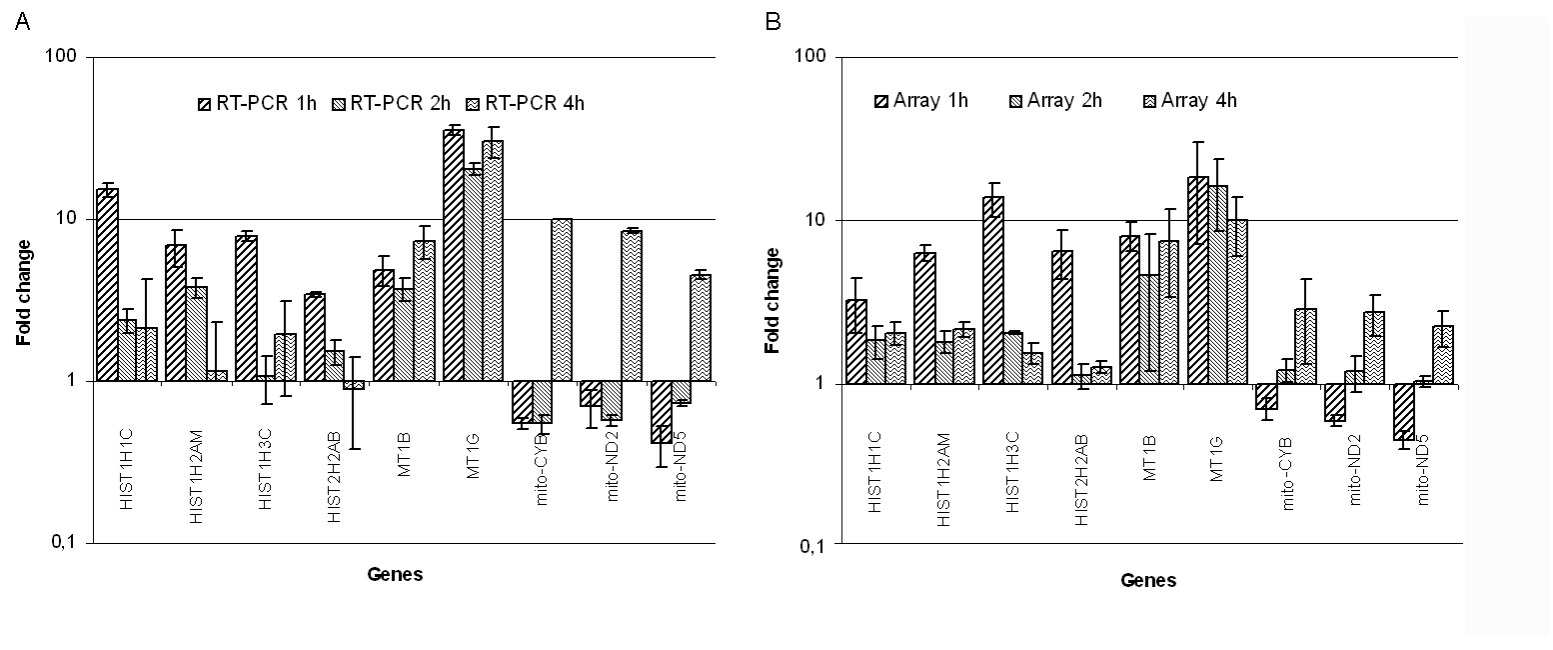
Real-time PCR-based verification of HAL-PDT effect. Nine selected genes – HIST1H1C, HIST1H2AM, HIST1H3C, HIST2H2AB, MT1B, MT1G, mitochondrial encoded genes (mito), mito-CYB, mito-ND2 and mito-ND5- were assessed for expression (relative mRNA level) at 1 h, 2 h and 4 h after HAL-PDT by real time PCR using the housekeeping RPLPO and TBP genes as reference genes. The fold change in gene expression (ΔΔCt) in photochemically treated samples compared to non-treated controls was calculated and presented as the mean ± St. Dev (A). The fold-changes obtained by the microarray analysis for the selected genes are presented for comparison. Fold-change of three repeated experiments presented as the mean ± St. Dev (B). Correlation factors for arrays and RT-PCR verified genes were 0.77; 0.99 and 0.85 for 1 h, 2 h and 4 h respectively.

Of the 170 probes detected as early genes (124 unique Entrez gene IDs), 53 genes were identified as involved in nucleosome assembly and belonged to the histone family of genes (Fig. 4). Interestingly, various subtypes of the linker histone, H1 (totally 49 histone 1 subtypes/forms), were affected to a high degree by HAL-PDT. Six different histone 2 subtypes/forms (HIST2H2AB, HIST2H2AC, HIST2H2BE, HIST2H2AA, HIST2H3C, HIST2H4) and histone 4, H4 (HIST4H4) were also up-regulated (Fig. 4). The complexing of histones with DNA, and the resulting condensation of chromatin, protects mammalian cell DNA from radiation-induced strand breakage [51]. Recently, it was reported that the ZnTPPS(4) sensitizer, when bound to cyclodextrin hpbeta CD, as used in PDT, can induce DNA breaks [52]. Also, beta-carboline derivatives (1,3,9-trisubstituted beta-carboline derivatives) were demonstrated to distribute within the nucleus and intercalate into DNA, causing direct DNA damage through photochemical reaction products in PDT [53]. Previously, it was assumed that ALA-mediated PDT might not induce a clear DNA-damage response, probably due to the fact that HAL-induced porphyrins are localized in the mitochondria and bio-membraneous structures. Since singlet oxygen, the main cytotoxic agent produced by light-activated sensitizers, has a lifetime of less than 0.05 μs, with a maximal diffusion of 0.02 μm from the site of its production [54], extra-nuclear components of the cells were originally expected to be the main targets of HAL-PDT. However, it was subsequently demonstrated that light activation of porphyrins derived from exogenous ALA produced DNA oxidative damage in a dose dependent manner [55-57]. The induction of histone genes might indicate a complexing of histones with DNA, with resulting condensation of chromatin to protect DNA from HAL-PDT caused by oxidative stress. The protective role of histones in chromatin condensation as a response to stress was recently challenged by Konishi at el [58], through the proposal of a novel mechanism for cells to sense a specific cellular stress with ignition of an apoptotic response to eliminate damage. A specific isoform of the linker histone, histone H1.2 (HIST1H1C), has been found to translocate from the nucleus into the cytoplasm, where it induces the release of cytochrome c from the mitochondria, with subsequent apoptosis of the cell [58]. While all nuclear histone H1 forms are released into the cytoplasm in a p53-dependent manner after X-ray irradiation, only H1.2, but not other H1 forms, induced cytochrome c release from isolated mitochondria in a Bak-dependent manner [58]. The HIST1H1C form was among the early up-regulated histone genes we found responsive to HAL-PDT. Many other histone 1 forms were affected by HAL-PDT, thus it is difficult to speculate if HIST1H1C is directly responsible of the deadly effect of HAL-PDT. A further pursuit of the effects of this isoform with respect to translocation and mechanism of action awaits future studies, as does a more detailed elucidation of the roles of various other histone members. The verification of three histone 1 genes (among them HIST1H1C) and one histone 2 gene showed good agreement between RT-PCR and microarrays (Fig. 5).

#### Metallothioneins

**Figure 6:**
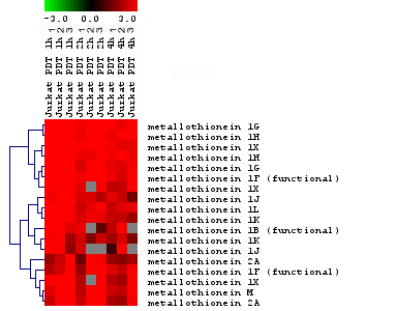
Cluster of metalothionein genes that were significantly altered in response to HAL-PDT. Horizontal stripes represent genes and columns show treatment protocols. The log2-fold changes of gene ratios are color coded as shown in the bar. The three different columns represent repeated microarray experiments.

The family of metallothioneins was highly represented as differentially expressed (10 genes) (Fig. 6). Metallothioneins (MTs) are a family of cysteine-rich proteins with high affinity for metals. MTs are known as scavengers of free oxygen radicals [59]. It has been shown that MTs may protect DNA from Cu-induced damage by free radicals through sequestering of copper, and thus prevents its participation in redox reactions [60]. Previous studies have also revealed that MTs can protect cells from apoptosis induced by oxidative stress and metals [59,61]. Since PDT-induced cellular damage is mainly due to free oxygen radicals [2,6], significant over-expression of the MT genes may indicate a protective role from the HAL-PDT-induced oxidative stress. This suggests that MTs may play a role in regulating apoptosis, and that modulation of MT expression may provide a strategy for altering cellular resistance to chemotherapeutic compounds [62] as well as PDT. The MT1B gene that was selected for verification showed good agreement between RT-PCR and arrays, while the expression of MT1G gene measured by arrays was somewhat lower than RT-PCR (Fig. 5).

### Late responses

Contrary to early responses, the number of altered genes/probes increased sharply to a total of 15,762 probes (10,373 unique Entrez gene IDs) at 4 h, clearly displaying the massive disturbances in the transcriptome within the pro-apoptotic cells. The proportion of genes with up-regulated expression decreased to 41.1% at 4 h, compared to 82.9% of up-regulated genes at early response. Since the destructive PDT dose likely causes high levels of oxidative stress, we cannot neglect that the high number of altered genes might likely indicate random transcriptome changes, caused by direct oxidation of non-specific mRNAs [63]. RNAs are reported to be more sensitive to photodynamic degradation than DNA [56], especially since most RNAs are cytoplasmic and exposed to higher ROS concentrations. In the case of RNA molecules themselves are photooxidative targets, one could expect that the mRNA transcripts would be damaged randomly and not bind to the specific probes on microarrays. As a result, the expression of genes will be measured as down regulated compare to untreated cells. To explore whether the observed RNA down-regulation at 4 h was direct and non-specific, or targeted, we performed a Gene Ontology and KEGG analysis. We observed that down-regulated genes contributed significantly in GO enriched groups within the total of differentially expressed genes (Table 1, 2). While up-regulated genes scored in other GO groups, a number of enriched extra-nuclear processes, especially mitochondrial, were present when down-regulated genes were scored. However, since HAL, the mitochondrial localizing sensitizer was used in the study [43,64-67], the direct effect on mitochondria was expected. Furthermore, enriched GO processes of up-regulated genes included both nuclear and extra-nuclear processes, such cytoskeleton, junctions and adhesion. Notably, because of the direct PDT effect to cytoskeleton, well-known house keeping proteins such actin or tubulin that is widely used for the quantitation of western data would not be representative controls. Thus, for western blots, the total protein content was used as a measure, by staining the gels after electrophoresis and signals normalized against total protein content. Interestingly, genes encoded proteins in the porphyrin metabolism were stimulated, indicating cellular response to excessive porphyrin production after the transfection with HAL, which is a heme precursor. Among these genes, 7 different UDP glucuronosyltransferase 1 and 2 family genes were induced. These are of major importance in heme degradation [68,69]. The gene ontology analysis suggested that the cell commitment to apoptosis was directly reflected in the transcriptome in a specific manner, and not random changes.

**Table 1:** Enriched KEGG pathways in the list of differentially expressed genes at 4 h after HAL-PDT

**KEGG Pathway**	**Gene Count**	**%**	P-Value*
**Total/Down regulated gene list**			
oxidative phosphorylation	101/79	1.0	9.1E-10
ribosome	125/119	1.2	2E-07
cell cycle	72/64	0.7	0.0015
citrate cycle (TCA cycle)	22/20	0.2	0.0041
proteasome	25/25	0.2	0.0043
ATP synthesis	32/27	0.3	0.0047
rna polymerase	21/20	0.2	0.0065
ubiquitin mediated proteolysis	32/26	0.3	0.0078
purine metabolism	92/72	0.9	0.011
pyrimidine metabolism	58/49	0.6	0.017
cholera – infection	28/20	0.3	0.02
glycolysis/gluconeogenesis	40/33	0.4	0.023
phenylalanine, tyrosine and tryptophan biosynthesis	10/8	0.1	0.027
propanoate metabolism	26/21	0.3	0.038
insulin signaling pathway	78/59	0.8	0.044
**Up-regulated gene list**			
ubiquinone biosynthesis	8	0.2	0.019
alzheimer's disease	10	0.2	0.022
neuroactive ligand-receptor interaction	74	1.5	0.027
type i diabetes mellitus	14	0.3	0.039
focal adhesion	54	1.1	0.044
fc epsilon ri signaling pathway	23	0.5	0.065
gap junction	27	0.5	0.072
long-term depression	22	0.4	0.076
adherens junction	22	0.4	0.085
porphyrin and chlorophyll metabolism	12	0.2	0.096
hematopoietic cell lineage	24	0.5	0.099

*The gene-enrichment of functional categories was measured by determining the number of genes, belonging to the functional group in the list of modulated genes weigh against to the total analysed genes on arrays (background) using Fisher Exact test.

**Table 2:** Enriched GOTERM Cellular Component in the list of differentially expressed genes at 4 h after HAL-PDT

**GOTERM Cellular Component (level 5)**	**Gene Count**	**%**	P-Value*
**Total/Down regulated gene list**			
mitochondrion	418/345	4.1	1.1E-17
mitochondrial envelope	124/104	1.2	2.2E-07
nuclear lumen	230/196	2.2	3.5E-07
mitochondrial membrane	113/94	1.1	1.3E-06
nucleus	2001/1390	19.5	1.3E-06
mitochondrial inner membrane	95/77	0.9	2.4E-06
mitochondrial lumen	56/50	0.5	7.7E-06
ribosome	223/200	2.2	0.000023
nucleolus	74/69	0.7	0.000035
large ribosomal subunit	41/34	0.4	0.00026
nucleoplasm	157/128	1.5	0.00062
cytosolic ribosome (sensu Eukaryota)	32/29	0.3	0.0007
mitochondrial electron transport chain	32/22	0.3	0.0007
cytosolic large ribosomal subunit (sensu Eukaryota)	23/20	0.2	0.00074
organellar ribosome	23/20	0.2	0.0018
mitochondrial ribosome	23/20	0.2	0.0018
heterogeneous nuclear ribonucleoprotein complex	15/15	0.1	0.0021
spliceosome complex	44/39	0.4	0.0039
small nucleolar ribonucleoprotein complex	21/21	0.2	0.0046
endoplasmic reticulum lumen	15/14	0.1	0.0063
proton-transporting ATP synthase complex (sensu Eukaryota)	12/9	0.1	0.011
endoplasmic reticulum	291/219	2.8	0.015
small nuclear ribonucleoprotein complex	13/13	0.1	0.017
proton-transporting two-sector ATPase complex	35/26	0.3	0.018
organellar small ribosomal subunit	12/11	0.1	0.029
endoplasmic reticulum membrane	55/47	0.5	0.035
**Up-regulated gene list**			
integral to plasma membrane	297	6.0	1.4E-06
intercellular junction	32	0.6	0.035
nucleosome	20	0.4	0.041
chromatin	38	0.8	0.044
actin cytoskeleton	47	0.9	0.14
nucleus	752	15.2	0.16
microsome	31	0.6	0.18
cytoskeleton	175	3.5	0.22
apical junction complex	15	0.3	0.33
microtubule associated complex	24	0.5	0.37
mitochondrial electron transport chain	10	0.2	0.4

*The gene-enrichment of functional categories was measured by determining the number of genes, belonging to the functional group in the list of modulated genes weigh against to the total analysed genes on arrays (background) using Fisher Exact test.

#### Oxidative stress/mitochondria

**Figure 7:**
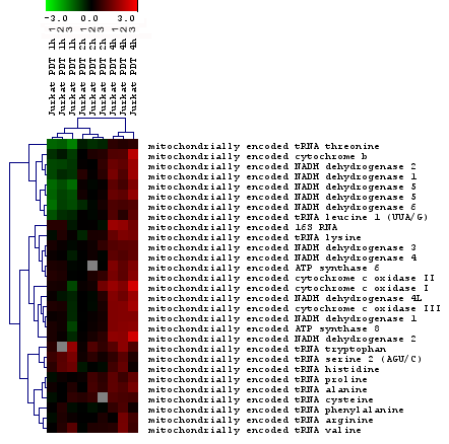
Cluster of mitochondria encoded genes that were significantly altered in response to HAL-PDT. Horizontal stripes represent genes and columns show treatment protocols. The log2-fold changes of gene ratios are color coded as shown in the bar. The three different columns represent repeated microarray experiments.

A total of 639 probes (418 unique Entrez gene IDs) from genes relevant to mitochondrial function were identified as altered, of which 78.1% were down-regulated. These genes encoded components of the mitochondrial envelope, the mitochondrial membrane, inter membrane, lumen and ribosome, as well as the proton-transporting ATPase complex. The mitochondrial localization of ALA or HAL-induced protoporphyrin-IX (PpIX) has been established [43,64-67]. The ultrastructural alterations of the mitochondria, in addition to higher percentages of cells losing the mitochondrial transmembrane potential, was reported following ALA-PDT [42,65,67]. A total of 101 genes altered by HAL-PDT were identified as being involved in oxidative phosphorylation (Table 1). This gene ontology group was the group most significantly (p = 9.1E-10) enriched among all gene groups. A total of 32 genes from the ATP synthesis coupled electron transport were found to be altered, the majority being down-regulated. This is consistent with previously published data, where ATP levels were found to be decreased at both 1 and 4 h after ALA-PDT [65]. Furthermore, dysregulation of the citrate cycle (TCA cycle) indicated that the cell energy balance was seriously damaged. Surprisingly, a majority of the mitochondrial nuclear encoded genes were found to be down-regulated, while mitochondrial encoded genes (23 genes) were found to be up-regulated at 4 h (Fig. 7). Mitochondria are the site of many essential biochemical reactions, an important subset of which requires proteins encoded in the mitochondrial DNA (mtDNA). How transcription of mtDNA is regulated in response to changing cellular demands is largely unknown. Previously, it has been reported that release of mitochondrial components can trigger an apoptotic response. However, it was reported that ATP depletion after mitochondrial photodamage does not play a role in initiation of the apoptotic program [70]. Previously, we reported that at 4 h following HAL-PDT nearly 80% of the cells exhibited typical apoptotic features and that PDT caused cytosolic translocation of cytochrome c and nuclear redistribution of apoptosis-inducing factor (AIF), followed by release of cytochrome c [43]. Here, we report that a mitochondrial response occurs also at the transcriptional level by repressing and inducing mitochondrially encoded genes. We found various NADH dehydrogenases to be downregulated at 1 h. Mitochondrially (mito-) encoded -CYB, mito-ND2 and mito-ND5 were verified by RT-PCR, and these were in agreement with array results (Fig. 5). Previously it has been reported that ALA-based PDT initiate two apoptotic processes proceeding in parallel, one representing the mitochondrial pathway, the other involving disruption of calcium homeostasis and activation of the endoplasmic reticulum stress-mediated pathway [25]. In our study, the calcium binding proteins (365 genes) had changed expression at 4 h post HAL-PDT, confirming the disruption of calcium homeostasis.

The combination of the cellular responses (induction of histones and metallothioneins) in addition to induction of mitochondrial encoded genes may indicate a partial repair of mitochondrial and other cellular functions. However, it is in general difficult to assess the impact of the changes in gene expression at 4 h, as cell mortality is the endpoint. One of the reasons could be damaged translational machinery, given that ribosomal proteins were identified as direct targets (Tables 1, 2).

#### Other pathways/genes

Other gene ontology processes found to be very significantly affected included those of the ribosome, cell cycle genes, proteosome, ubiquitin mediated proteolysis (Table 1), as well as protein transport, and ER to Golgi vesicle mediated transport (data not shown). This indicated that main cellular processes, such as the cell cycle and protein synthesis/transport were disrupted, leading to protein degradation through ubiquitine mediated proteolysis. In addition, the processes such pyrimidine and purine metabolism, glycolysis/gluconeogenesis, RNA/DNA metabolism, cellular protein metabolism, protein biosynthesis scored highly among enriched gene ontology groups indicating disruption of these processes. This seems to be an indication that the basic energy metabolism is shutting down, or at least is heavily affected, as one should expect from apoptotic cells.

### Effect on alternative splicing

**Figure 8:**
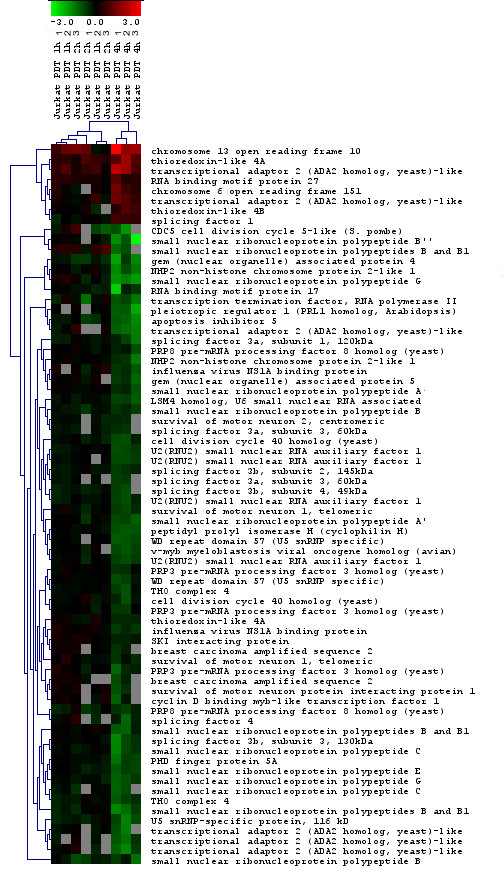
Cluster of spliceosome complex genes that were significantly altered in response to HAL-PDT. Horizontal stripes represent genes and columns show treatment protocols. The log2-fold changes of gene ratios are color coded as shown in the bar. The three different columns represent repeated microarray experiments.

In our experiments, we observed that HAL-PDT resulted in expression changes in the spliceosome complex. A total of 72 probes (44 unique Entrez gene IDs) showed significant changes at 4 h (Table 2), while expression of 39 genes were down-regulated (Fig. 8). None of the splicing factors were significantly altered at early response time points. Alternative splicing is tightly regulated in a tissue-specific and developmental-stage-specific manner [30,71]. Since the microarrays included constitutive and alternative splice variant probes, we examined how many genes were alternatively spliced in response to HAL-PDT (Table 3). A total list of altered splice variants is presented [see Additional file 1]. Interestingly, of 32 genes with altered expression, 24 alternatively spliced variants were down-regulated already after 1 h. Notably, we did not detect changes in the transcriptional level of splice factors at early response times. However, stress-induced signaling mechanisms that regulate pre-mRNA splicing *in vivo*, by influencing the subcellular distribution of splicing factors, have been described [72]. This type of the regulation will not be detected by monitoring the transcription of splice factors. The increased proportion of down-regulated alternatively spliced variants remained similar (70.6 and 69.2%) at 2 and 4 h, most likely signifying a dysfunction of the splicing machinery following treatment. Furthermore, when the low fold changed variants [1–1.99] were taken into account, the percent of down-regulated splice variants increased to 90, 80 and 65.6, at 1, 2 and 4 h post HAL-PDT, respectively. In contrast, in the groups of the alternative splice variants altered by higher fold change [≥2], the proportion of up- and down-regulated genes were close to equal (Table 3). This indicated nonrandom (caused by the dysfunction of splicing machinery) changes in alternative splicing. Thus, we used GO to uncover whether the genes with alternatively spliced variants could be mapped to particular GO groups/altered processes (see Table 4 for GO biological processes of altered splice variants at 4 h). Interestingly, the GO processes of altered alternative splice variants were relatively similar to the previously scored GO groups, and included different metabolic processes, apoptosis, cell cycle as well as transcription. Interestingly, 5 genes had up-regulated, and 37 had down-regulated alternative splice variants of the genes that had transcription factor activity. Notably, alternative splicing of pre-mRNAs encoding transcription factors is one of the common mechanisms for generating the complexity and diversity of gene regulation [73]. One of the genes was identified as activating transcription factor 3 (ATF3), that had an induced alternative splice variant at all three studies time points, while the constitutive, full-length variant was low expressed and did not change with the treatment. ATF3 is a member of the mammalian activation transcription factor/cAMP responsive element-binding (CREB) protein family of transcription factors [74]. ATF3 plays a role in determining cell fate and has been shown to generate a variety of alternatively spliced isoforms in response to stress [75-78]. It was demonstrated that ATF3 (deltaZip2) isoform, but not full-length ATF3, sensitizes cells to apoptotic cell death, partly by suppressing the expression of the NF-kappaB-dependent anti-apoptotic genes cIAP2 and XIAP. ATF3 (deltaZip2) has further been shown to bind directly to the p65 subunit of NF-kapaB and down-regulate the CBP/p300 recruitment complex [78]. The sequence analysis of the detected alternative splice variant revealed that the alternative splice variant induced by HAL-PDT was the ATF3 isoform (deltaZip2), demonstrating a direct cell fate control through alternative splicing in HAL-PDT response. Thus, the results stress the importance of alternative splice variant mapping. If not performed, this may lead to false interpretations of the results.

**Table 3:** Counts of alternative splice variants

**Filtering criteria**	**1 h**	**2 h**	**4 h**
Number of alternative spliced genes, cut off fold change ≥1	34	13	442
Number of alternative spliced genes, cut off fold change ≥2	14	2	256
Number of alternative spliced genes, cut off fold change [1–1.99]	20	10	192
			
Upregulated alternative variants, cut off fold change ≥1	10	2	125
Upregulated alternative variants, cut off fold change ≥ 2	7	2	101
Upregulated alternative variants, cut off fold change [1–1.99]	3	1	27
			
Dowregulated alternative variants, cut off fold change ≥ 1	24	9	261
Dowregulated alternative variants, cut off fold change ≥ 2	6	1	138
Dowregulated alternative variants, cut off fold change [1–1.99]	18	8	126
			
Alternatives up and down for the same gene, cut off fold change ≥ 1	0	2	56
Alternatives up and down for the same gene, cut off fold change [1–1.99]	0	0	17

% of down regulated alternatives, cut off fold change ≥ 1	70.6	69.2	59.0
% of down regulated alternatives, cut off fold change ≥ 2	42.9	50.0	53.9
% of down regulated alternatives, cut off fold change [1–1.99]	90.0	80.0	65.6

**Table 4:** Enriched GOTERM Biological Process in the list of alternatively spliced genes

**Up-regulated splice variants, with fold change [≥1] at 4 h**	**Gene Count**	**%**	P-Value*
regulation of protein metabolism	5	5.7	0.029
cellular protein metabolism	24	27.3	0.041
phosphate metabolism	9	10.2	0.097
cytoskeleton organization and biogenesis	5	5.7	0.16
RNA metabolism	5	5.7	0.23
biopolymer modification	12	13.6	0.27
macromolecule biosynthesis	6	6.8	0.49
**Down regulated splice variants, with fold change [≥1] at 4 h**			
cellular lipid metabolism	15	6.4	0.0094
negative regulation of cellular metabolism	8	3.4	0.035
membrane lipid metabolism	6	2.6	0.042
regulation of progression through cell cycle	13	5.5	0.044
intracellular transport	15	6.4	0.058
biopolymer modification	33	14.0	0.06
establishment of cellular localization	15	6.4	0.063
DNA metabolism	15	6.4	0.073
amino acid metabolism	8	3.4	0.073
protein catabolism	7	3.0	0.1
intracellular protein transport	9	3.8	0.1
biopolymer catabolism	7	3.0	0.13
regulation of protein kinase activity	5	2.1	0.15
carboxylic acid metabolism	11	4.7	0.15
endocytosis	5	2.1	0.17
cofactor biosynthesis	5	2.1	0.18
steroid metabolism	5	2.1	0.18
negative regulation of cell proliferation	5	2.1	0.2
lipid biosynthesis	6	2.6	0.22
regulation of nucleobase, nucleoside, nucleotide and nucleic acid metabolism	37	15.7	0.26
apoptosis	11	4.7	0.31
transcription	37	15.7	0.33
cellular macromolecule catabolism	7	3.0	0.36
protein complex assembly	6	2.6	0.4
protein transport	10	4.3	0.44
chromosome organization and biogenesis	6	2.6	0.44
vesicle-mediated transport	7	3.0	0.45
synaptic transmission	5	2.1	0.46

*The gene-enrichment of functional categories was measured by determining the number of genes, belonging to the functional group in the list of modulated genes weigh against to the total analysed genes on arrays (background) using Fisher Exact test.

### Correspondence between alternative microarray platforms: cDNA and oligo microarrays

Molecular confirmation of microarray results is important when checking for consistencies of expression measurements using different methods. RT-PCR and Western blotting were used for selected genes. Recently, different microarray platforms have been reported to have a good agreement [79,80]. Therefore, we included experiments on one of the time points (1 h) performed on cDNA microarrays. In agreement with oligo microarrays, top ranking of the genes showed alteration in ATF3, CERBPB, JUN, JUND, IER2, as well as metallothioneins and histone coding genes (Table 5).

**Table 5:** Ranking cDNA and Oligo microarray detected genes

Locus Link	Gene Symbol	Gene Name	Heebo Rank	cDNA Rank
467	ATF3	Activating transcription factor 3	16	7
4490	MT1B	Metallothionein 1B (functional)	29	152
8349	HIST2H2BE	Histone 2, H2be	41	180
3725	JUN	V-jun sarcoma virus 17 oncogene homolog (avian)	44	60
3727	JUND	Jun D proto-oncogene	51	20
9592	IER2	Immediate early response 2	65	154
8334	HIST1H2AC	Histone 1, H2ac	77	41
3021	H3F3B	H3 histone, family 3B (H3.3B)	113	97
4495	MT1G	metallothionein 1G	116	158
3006	HIST1H1C	Histone 1, H1c	266	128
2781	GNAZ	Guanine nucleotide binding protein (G protein), alpha z polypeptide	282	72
1051	CEBPB	CCAAT/enhancer binding protein (C/EBP), beta	295	18
6428	SFRS3	Splicing factor, arginine/serine-rich 3	331	67
10957	PNRC1	Proline-rich nuclear receptor coactivator 1	342	84
5366	PMAIP1	phorbol-12-myristate-13-acetate-induced protein 1	354	21
3251	HPRT1	Hypoanthine phosphoribosyltransferase 1 (Lesch-Nyhan syndrome)	530	176
168374	ZNF92	Zinc finger protein 92 (HTF12)	542	33
7128	TNFAIP3	Tumor necrosis factor, alpha-induced protein 3	596	134
5475	PPEF1	Protein phosphatase, EF hand calcium-binding domain 1	731	153
58483	C9orf27	Chromosome 9 open reading frame 27	766	146

## Conclusion

In summary, the results provided new insights into how a PDT relevant dose alters gene expression. We confirmed known early defense response genes, and extended these to include the involvement also of other oxidative stress inducible gene groups, most notably the metallothioneins and histones. We also demonstrated that high levels of cellular cytotoxicity had direct effects on nuclear and mitochondrial DNA transcription levels in impairing metabolic processes. Furthermore, we attempted to identify how different signaling processes may be involved in the execution of cell death. The results indicated that mitochondrial damage caused by HAL-PDT modulates alternative splicing through unbalancing a vast number of isoform equilibriums, which may be an important contribution to the deadly outcome of HAL-PDT therapy.

## Methods

### Cell culture and PDT treatment

The human T-cell leukemia cell line, Jurkat, was maintained in RPMI 1640 medium containing 10% fetal calf serum (FCS), 100 U/ml penicillin, 100 μg/ml streptomycin and 1% glutamine in a fully humidified incubator (Nuaire US Autoflow) at 37°C with 5% CO_2_. In experiments with PDT, 8 × 10^5^/ml of cells were seeded in 6-well plastic tissue-culture plates (Nunc) and incubated in the dark for 4 h in serum-free RPMI 1640 medium containing 5 μM of hexaminolevulinate (HAL) (PhotoCure ASA, Oslo, Norway).

Since the light penetration is undemanding for the cell line, the light source used in the present study has a broad spectrum with a range of 400–500 nm, fitting largely to the maximal absorption of porphyrins derived from ALA and its esters including HAL. Our previous studies have shown that the combination of HAL with the light source can kill various types of tumor cells efficiently [42,43]. The cells were exposed to light from a bank of four fluorescent tubes (model 3026, Applied Photophysics, London, UK). The fluence rate of the light reaching the cells was 8 mW/cm^2^. The light doses of 80, 160 and 240 mJ/cm^2 ^were used to determine the cell viability. The light dose of 160 mJ/cm^2 ^was chosen to treat the cells used in microarray experiments. The cells were washed twice with medium immediately after illumination and incubated with fresh medium containing 10% FCS. Cells were washed twice with PBS before sampling.

### Cell survival

Cell survival was measured by the MTS assay [81]. After HAL-PDT, 100 μl of each sample was placed to 96-well plastic microplates (Nunc) and incubated at 37°C for 0, 1 and 3 h, followed by the addition of 20 μl of MTS (5 mg/ml) (Promega Corporation, Madisom, WI, USA) into each well for an additional 1 h-incubation. The absorbance at 490 nm was measured with a microplate reader (Labsystems Oy, Helsinki, Finland). The absorbance of blank wells containing medium and MTS, but no cells, was subtracted from all readings and cell survival was expressed as the fraction of control samples.

### RNA purification and labelling

Total RNA was isolated using GenElute Mammalian Total RNA kit (Sigma, St. Louis, MO) according to the manufacturer's protocol. RNA concentrations were determined with NanoDrop spectrophotometer (NanoDrop Technologies). The integrity and degree of degradation of RNAs was calculated using Agilent 2100 Bioanalyzer (Agilent Technologies AS). Fluorescence-labelled cDNAs were synthesized from 20 μg of the total RNA using an indirect amino allyl microarray labelling kit (Faiplay, Stratagene, La Jolla, CA, USA) according to the manufacturer's recommendations.

### Oligo microarrays and their hybridization

The oligo microarrays used in this study were obtained from The Norwegian Microarray Consortium, for details on the arrays, we refer to [82]. The target oligos were the HEEBO (Human Exonic Evidence Based Oligonucleotide) set (Invitrogen). Probes included probes for constitutive, alternative exonic; alternative spliced/skipped exons, mitochondrial genes (mtDNA) for more details on oligos, we refer to [83]. During analysis all probes were handled individually, also those that encoded the same gene. The microarray hybridization buffer (76 ul) contained each of the labeled probes, 16 μg poly A (Amersham Pharmacia Biotech AB), 8 μg yeast tRNA, 0.5 μg herring's sperm DNA, 5 × SSC, 0,1% SDS, 25% (v/v) formamid. Prior to hybridization, the solution was incubated for 2 min at 100°C and then centrifuged for 2 min at 13,000. The microarray slides were hybridised at 42°C overnight in a humid hybridization chamber (TeleChem International, Inc.). The slides were washed for twice in 0.5× SSC, 0.01% SDS, and 0.06× SSC for 5 min at room temperature. The slides were spun and dried immediately after washing.

The microarray experiment design was based on competitive hybridizations of HAL-PDT treated cells vs untreated. Total RNA was harvested from triplicate samples of treated and non-treated cells at 1, 2 and 4 h after HAL-PDT. Three replicated hybridization were performed for each time point.

### cDNA microarays and their hybridization

The cDNA microarrays used in this study were obtained from The Norwegian Microarray Consortium, for details on the arrays, we refer to [82]. The targets, approximately 15.000 unique I.M.A.G.E cDNA clones were from ResGen 40 k set. The hybridization volume of 110 μl consisted of: 8–10 μl of each of the labelled probes, 16 μg poly A (Amersham Biosciences), 15 μg human Cot-1 DNA (Invitrogen) and 85–90 μl of Microarray Hybridization Buffer #1 from Ambion (Austin, TX). The final mix was heated for 2 min at 100°C and after spinning down it was applied on a microarray. A hybridization station (Genomic Solutions, Inc., Ann Arbor, MI) was used for hybridization and wash, the details on hybridizations and wash can be found [82].

### Data preparation and analysis

Slides were scanned using an Agilent Microarray scanner (Agilent Biotechnologies). The quantitative measurements of the fluorescence images were performed by the software GenePix 4000B (Axon Instruments, Union City, CA). The images (TIFF files) and extracted raw data (GRP files) were stored in a BASE 1.2.15 database [84]. The spots that were technically flawed or flagged automatically by the GenePix software, spots with a diameter less than 60 μm were removed from the data of each microarray. The genes were preserved signal-to-noise ratio in one of the channel was equal or higher then 3 removing uncertain spots. Background-subtracted intensities less than one times the sdtev of the local background were assigned this value to avoid zero or negative values in the ratio calculations. Moreover, systematic errors were corrected by normalizing the data using a locally weighted scatter plot smoother, the method of pin-based lowess[85] The genes were preserved if the values were experimentally obtained in more than 70% of the experimental matrix. The weighted K-nearest neighbours method was applied for imputation of missing values [86].

We used Limma package in R [87] to define differentially expressed genes. As we had three groups (1, 2 and 4 h) and three replicates in each group, the linear model fitting and empirical Bayes methods were used for assessing differential expression. The empirical Bayes approach is equivalent to shrinkage of the estimated sample variances towards a pooled estimate, resulting in far more stable inference when the number of arrays is small [88]. To classify a series of related t-statistics as up, down or not significant, the multiple testing across genes and contrasts (1, 2 and 4 h groups) was used. To avoid the multiple testing problem [89] we have used Benjamine and Hochberg's method [90] to control the false discovery rate across the genes with restriction p < 0.05. The venn diagrams were prepared with Limma to visualize the intersections of the significant gene sets within groups. Hierarchical clustering of genes for visualisation of expression patterns was performed in MultiExperiment Viewer (MEV) [91]. Functional classification was performed by using the Database for Annotation, Visualization and Integrated Discovery (DAVID, release 2.1) online tools. The relative enrichment of KEGG pathways (KEGG PATHWAY Database (July 19, 2006)) was calculated the number of genes, belonging to these functional categories in the list of significantly altered genes. The gene-enrichment of functional pathways measured by determining the number of genes, belonging to the pathway in the list of significantly altered genes weigh against to the total analysed/printed genes on arrays (background) using Fisher Exact test. For top raking of the genes, only 1 h post HAL-PDT experiments performed cDNA and oligo arrays were taken. The join was done using locus link IDs, so a gene appears as ranked only if locus links IDs present for both the cDNA and oligo significantly altered genes. Since many oligo probes map to the same locus link, the highest-ranking oligo probe for a particular locus link is used in the ranking.

### Gel electrophoresis and immunoblotting

For protein isolation, the cell pellets were lysed on ice in 1 ml of lysis buffer (0.4% w/v SDS, 5 mM EDTA, 5 mM EGTA, 10 mM sodium pyrophosphate and 20 mM Tris-base; pH 7.2) for 30 min, briefly sonicated, diluted 1:2 in double strength SDS gel-loading buffer [double strength, 1% (w/v) SDS, 4.8 mM sodium deoxycholate, 10% (v/v) mercaptoethanol, 1% (v/v) Igepal CA-630, ~0.1% (w/v) Bromophenol Blue, 13,4% (v/v) glycerol and 120 mM Tris/HCl, pH 6.8] and boiled for 5 min at 95°C. After measuring the protein contents of the extracts with the BCA protein assay kit from Pierce (Rockford, USA) [92], 10 μg of samples were separated by SDS gel electrophoresis for 40 min at 200 V in 10% polyacrylamide gels containing 0.1% SDS. Molecular weight markers were included in all gels. Gel-separated proteins were transferred to nitrocellulose blotting membranes using a semi-dry transfer unit (Bio-Rad Laboratories, Hercules, CA, USA) with Towbin's blotting buffer (192 mM glycine, 20% methanol and 25 mM Tris-base; pH 8.3). The membranes were blocked by overnight incubation with 5% dry milk in TBS containing 0.2% Tween-20 (TBS-T) at 4°C, and washed three times for 10 min each in TBS-T. For detection of proteins, the membranes were incubated with the antibodies of MYC, ATF3 and JUN (diluted 1:1000 in TBS-T) overnight at 4°C, washed three times with TBS-T and incubated with the respective anti-rabbit or mouse-horseradish peroxidase-conjugated secondary antibodies (diluted 1:2000 in TBS-T) for 1 h at room temperature before being visualized by chemiluminescence using an ECL Western Detection Kit (Amersham Biosciences). To verify optimal blotting condition, the remaining proteins in the polyacrylamide gels were routinely stained with coomassie solution (0.1% (w/v) Coomassie blue R350, 20% (v/v) methanol, and 10% (v/v) acetic acid) for 2 hours and excess staining was removed by using a destaining solution (50% (v/v) methanol in water with 10% (v/v) acetic acid) over night.

### RT-PCR

The same source of total RNAs (as in the microarray experiments) was used for real-time RT-PCR validation of selected genes. cDNA was synthesized from 1 μg DNase I-treated RNA using the iScript cDNA synthesis kit (Bio-Rad, Hercules, CA). The cDNA solution was diluted 1:3 or 1:6 with nuclease-free water. Real-time PCR was performed using the iQ SYBR Green supermix (Bio-Rad) and specific primer pairs for the selected genes (specified in Fig. 5 and Table 6). Primers were designed using the software Primer Express 2.0 (Applied Biosystems, Foster City, CA). For each sample the following mix (was prepared: 10 μl cDNA, 30 μl iQ SYBRGreen Supermix, 300 nM of each primer (MWG Biotech AG, Germany) and nuclease-free water to a final volume of 60 μl. Aliquots of 25 μl were distributed in two wells on the PCR plate. Real-time PCR reactions were run on an iCycler (Bio-Rad) with the following amplification protocol: 3 min initial denaturation at 95°C, 50 cycles of 10 s denaturation at 95°C and 35 s annealing/extension at 60°C. A final melt curve analysis was included to verify that one specific product was obtained in each reaction. The fold changes in the relative gene expression were calculated using the Gene Expression Macro, version 1.1 (Bio-Rad). Calculations were based on the ΔΔCt method, in which the threshold cycle number (Ct) for each studied gene in each sample were normalised to the Ct-value of the reference gene in the same sample. As a reference gene we used human acidic ribosomal phosphoprotein P0 (RPLPO) and TATA binding protein (TBP). Both refrence genes were tested and showed not to be affected by the photodynamic treatment. The normalized values (ΔCt) in the control sample were given value of 1, and the fold-change between the photodynamic treated samples and the controls were calculated accordingly.

**Table 6:** Primers for RT-PCR

Gene Symbol	Primer	
HIST1H2AM	Forward primer:	5' TAGGCCACAGGTCGTTTTACCA
	Reverse primer:	5' GGAAATTGGAGCCCAGCTCTAG
HIST1H3C	Forward primer:	5' AAGTCCACCGAGCTGCTGAT
	Reverse primer:	5' AACGCAGGTCGGTTTTGAAG
HIST2H2AB	Forward primer:	5' CATCATCCCTCGCCATCTG
	Reverse primer:	5' AATGGTGACACCCCCGAGTA
MT1B	Forward primer:	5' GCAAGAAGTGCTGCTGCTCTT
	Reverse primer:	5' TGATGAGCCTTTGCAGACACA
MT1G	Forward primer:	5' CCTGTGCCGCTGGTGTCT
	Reverse primer:	5' TGCAGCCTTGGGCACACT
HIST1H1C	Forward primer:	5' GCGGCCACTGTAACCAAGA
	Reverse primer:	5' AGCAGCACTTTTGGCAGCTT
MT-ND5	Forward primer:	5' GATGATACGCCCGAGCAGAT
	Reverse primer:	5' AGGCGAGGATGAAACCGATAT
MT-ND2	Forward primer:	5' TCCAGCACCACGACCCTACT
	Reverse primer:	5' TTCGATAATGGCCCATTTGG
MT-CYB	Forward primer:	5' CCCTAGCCAACCCCTTAAACA
	Reverse primer:	5' GGACGGATCGGAGAATTGTG

### Splicing analysis

A program was written to create a concordance between locuslink IDs and HEEBO constitutive and alternative exon probes, using annotation information provided with the HEEBO probe set. This concordance grouped each locuslink ID with the oligo IDs of the probes targeting the exons contained within the locus.

For a each locus L, we applied a test to determine if there was a difference in expressed spliced isoforms between the treated and reference sample. At a hybridization at a given timepoint, we searched for at least one probe A derived from an alternatively expressed exon in L such that 1) The absolute value of log2ratio (A) was above a given threshold (fold change of PDT treated vs. none treated controls by 1 and 2) there existed a probe C in L from derived from a constitutive exon such that the absolute value of the difference between the log2ratios of A and C was equal to or greater than 1. Thus, for each hybridization, we found a (possibly empty) set of alternatively-expressed exon probes for each locus L. Since we performed three hybridizations are each timepoint, we required at least two of the three to agree in their results. Specifically, we required that there be a non-empty intersection between the set of alternatively-expressed exon probes from at least two experiments at the timepoint.

## Abbreviations

PDT, photodynamic therapy; HAL, hexaminolevulinate; ALA, 5-aminolevulinic acid; FDR, false discovery rate, GO; gene ontology; ROS, reactive oxygen species; KEGG, Kyoto Encyclopedia of Genes and Genomes.

## Authors' contributions

LC performed microarrays, carried out the statistical and GO analysis, drafted and edited the manuscript. QP responsible for the study design regarding PDT performance, contributed in manuscript revision. AR carried out splicing and ranking analysis. SS run PDT experiments. ST planned RT-PCR experiments, designed primers, tested housekeeping genes and helped with RT-PCR analysis. IEF performed cell viability after PDT tests. EH conceived the study, guided the practical work, data analysis and revised the manuscript.

All authors read and approved the final manuscript.

## Supplementary Material

Additional file 1 A total list of altered splice variants after HAL-PDT. The data provided represent the modulated alternative variants after 1, 2 and 4 h post HAL-PDT. Click here for file
